# Transforming precision medicine: The potential of the clinical artificial intelligent single‐cell framework

**DOI:** 10.1002/ctm2.70096

**Published:** 2025-01-06

**Authors:** Christian Baumgartner, Dagmar Brislinger

**Affiliations:** ^1^ Institute of Health Care Engineering with European Testing Center of Medical Devices Graz University of Technology Graz Austria; ^2^ Department of Cell Biology Histology and Embryology Gottfried Schatz Research Center Medical University of Graz Graz Austria

**Keywords:** Artificial Intelligence, Digital Cell Twins, Precision Medicine, Predictive Diagnostics, Regulatory Approval, Single-Cell Informatics

## Abstract

The editorial, “Clinical and translational mode of single‐cell measurements: An artificial intelligent single‐cell,” introduces the innovative clinical artificial intelligence single‐cell (caiSC) system, which merges AI with single‐cell informatics to advance real‐time diagnostics, disease monitoring, and treatment prediction. By combining clinical data and multimodal molecular inputs, caiSC facilitates personalized medicine, promising enhanced diagnostic precision and tailored therapeutic approaches. Despite its potential, caiSC lacks comprehensive data coverage across cell types and diseases, presenting challenges in data quality and model robustness. The article explores development strategies such as data expansion, machine learning advancements, and interpretability improvements. Future applications of caiSC could include digital cell twins, offering in‐depth simulations of cellular behavior to support drug discovery and personalized treatments. Regulatory considerations are discussed, underscoring the need for SaMD/AIaMD certifications for clinical use. Ultimately, with further refinement, caiSC could transform clinical decision‐making, driving personalized, precision medicine, and improved patient outcomes.

**Key points:**

- Integration of AI with Single‐Cell Informatics for Precision Medicine: The caiSC system combines artificial intelligence and single‐cell data to improve diagnostics, treatment predictions, and personalized medical decision‐making.
- Challenges in Data Coverage and Model Robustness: caiSC currently faces limitations due to incomplete data across cell types, diseases, and organs, as well as challenges in data quality and high computational demands, which affect model accuracy and clinical applicability.
- Future Potential and Regulatory Needs: The caiSC framework's development could lead to innovations such as digital cell twins, enabling personalized simulations of cellular responses for better treatment planning, though regulatory certification is essential for safe clinical use.

## 1 INTRODUCTION

**FIGURE 1:**
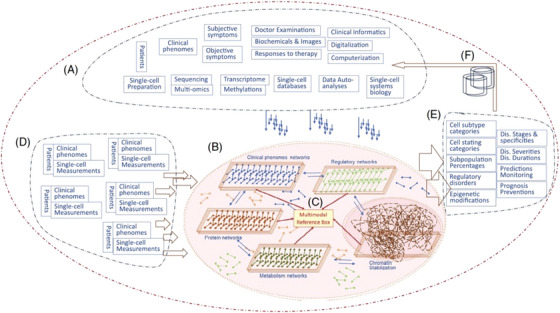
A schematic representation of the six critical sections of the clinical artificial intelligent single‐cell. (A) Dynamic generator of clinical single‐cell informatics as a source of clinical and molecular phenomes. (B) Artificial intelligent single cell with various reference boxes as an operator of comprehensive data analyses and integrations. (C) Intercommunication among multimodal reference boxes as a centre of multi/trans‐omic data crossing. (D) The input of clinical and molecular phenomes from the individual at the Department of Clinical Hematology & Biochemicals. (E) The outputs from the artificially intelligent single‐cell as the representatives of clinical outcomes. (F) The machine‐learning system with the capacity of re‐storing and remembering.^1^

The recently published editorial “Clinical and translational mode of single‐cell measurements: An artificial intelligent single‐cell” introduces a pioneering knowledge base concept known as the clinical artificial intelligence single‐cell (caiSC) system.^1^ This platform combines advanced artificial intelligence (AI) technologies with single‐cell informatics to deliver real‐time diagnostics, disease monitoring, and treatment predictions, thereby enhancing personalized medical decision‐making and prognostic strategies. By integrating clinical data, multimodal molecular inputs and AI‐driven analysis, caiSC aims to bridge the divide between single‐cell biology and clinical applications. This approach promises more precise diagnoses and personalized treatments with the potential to significantly improve patient outcomes (Figure 1).

Despite its transformative potential, the caiSC platform is not yet a fully comprehensive knowledge base encompassing data from single‐cell systems biology, organs, and their associated pathologies and diseases. This commentary explores how caiSC can provide valuable clinical insights and examines the key challenges, limitations, and future development strategies that need to be addressed for it to reach its full potential.

### 1.1 Challenges and development strategies for caiSC

Although caiSC currently lacks exhaustive data collection across a wide range of single‐cell data, pathologies and diseases, it remains a valuable tool for hypothesis generation and predictive modelling using available single‐cell and multi‐omics data. By analyzing patterns and correlations within these datasets, machine learning (ML) and AI‐based models in the caiSC framework can infer missing data through probabilistic methods. These models can also target specific disease pathways or cellular subsystems, providing meaningful insights even when data is sparse.^2^

For example, despite incomplete datasets, caiSC can generate personalized treatment recommendations or prognoses by identifying critical biomarkers in individual patients. The platform can also simulate disease progression or drug responses based on current knowledge, enabling predictive modelling even for less‐understood diseases. ML techniques, like transfer learning, further enhance caiSC's adaptability, allowing it to apply insights across various cell types or diseases, thereby improving predictions in underrepresented areas. This approach has the potential to yield insights into previously unidentified cellular mechanisms, advancing research in diverse fields such as cancer biology, immunology, or regenerative medicine.

However, caiSC faces several challenges, including uneven data availability across cell types, organ systems and diseases. This disparity leads to stronger models for well‐studied areas, while less‐researched fields present gaps in the system's coverage. Furthermore, data quality varies due to factors such as experimental noise or batch effects, which hinder accurate modelling. The complexity of single‐cell data, with its high‐dimensional interactions between genes, proteins and metabolites, poses significant challenges for comprehensive modelling, particularly when microenvironmental factors or disease states introduce additional variables.

Addressing these issues will require continued collaboration with research institutions and the integration of public single‐cell data initiatives, such as the Human Cell Atlas.^3^ Expanding datasets and standardizing data formats will enhance the system's accuracy and usability. Advanced AI and ML techniques, including unsupervised and semi‐supervised learning, can extract insights even from incomplete or unannotated datasets, helping to bridge existing knowledge gaps. Active learning, where the system suggests areas for further experimentation, will create feedback loops that gradually refine models.^4^

Future strategies should also prioritize enhancing the interpretability of caiSC outputs for clinicians, ensuring that AI‐driven insights are actionable. Ethical considerations must be integral to the system's development, with a particular focus on data privacy, bias reduction, and transparency. Achieving regulatory approval for ML/AI‐driven clinical decision‐making tools is crucial to building trust, ensuring legal compliance and safe implementation in clinical practice.

### 1.2 Potential for developing whole‐cell models and digital twins

One of the most promising applications of the caiSC framework is its capacity to develop functional or phenomenological whole‐cell models, often referred to as digital twins.^5^ Whole‐cell models aim to simulate the complete behaviour of a cell or its components by integrating multi‐omics, bioelectric and other types of data, providing a comprehensive view of cellular processes. These models can capture the complex interactions between genes, proteins and environmental factors, allowing for simulations of cellular behaviour under both normal and diseased conditions. By creating digital twins that mirror phenotypic data and therapeutic responses, caiSC has the potential to revolutionize drug discovery and personalized medicine.^6^ These digital twins could simulate disease progression, immune responses or drug effects at the cellular level, providing clinicians with predictive tools for patient‐specific treatment planning. For instance, a digital twin of a patient's cells could simulate their response to a new drug, predicting potential resistance or side effects. These models could play a critical role in understanding disease pathology and advancing therapeutics, allowing for in silico testing of treatment strategies before clinical trials. However, achieving the required accuracy and reliability while maintaining usability for clinicians remains a significant challenge.

The complexity and level of abstraction required for whole‐cell models depend on the available data and their intended application. Simplified models might be valuable for drug discovery or predicting therapeutic responses at the cellular level, while advanced models could simulate cell subsystem behaviour in complex conditions such as cancer metastasis or immune responses to infection. However, challenges such as high computational demands and the need for comprehensive, high‐quality data persist. To make whole‐cell models clinically viable, ongoing improvements in computational power, data standardization, and model interpretability are essential.

### 1.3 Regulatory considerations for ML/AI‐driven medical tools

When computer‐aided decision‐making tools, such as those powered by the caiSC framework, are used exclusively for research, regulatory approval is not required. This allows for greater flexibility in the technical development and potential applications of caiSC. However, clinical use of these tools mandates certification as Software as a Medical Device (SaMD) or AI as a Medical Device, in line with international regulatory standards.

Regulatory pathways for SaMD, including AI‐driven algorithms, have been outlined by organizations such as the US Food and Drug Administration and other international authorities.^7^, ^8^, ^9^ Despite these guidelines, challenges persist in the approval of ML‐ and AI‐based systems for clinical use. A major issue is the certifiability of AI systems, particularly dynamic, self‐learning algorithms. Static AI models, which remain unchanged after deployment, are easier to validate and certify. In contrast, dynamic AI systems that adapt over time pose significant challenges in maintaining continuous verification and validation required for clinical safety.

Achieving regulatory approval for these systems requires transparent model validation, extensive clinical trials, and continuous oversight. These measures are essential to ensure AI‐driven tools based on caiSC are safe, reliable, and effective in clinical settings. Regulatory approval will be crucial for enabling wider adoption and fostering trust in AI‐driven medical decision‐making. Furthermore, standardization of procedures and terminology within the caiSC framework will be essential for ensuring clear communication and seamless integration across various medical domains.

## 2 CONCLUSION

**FIGURE 2:**
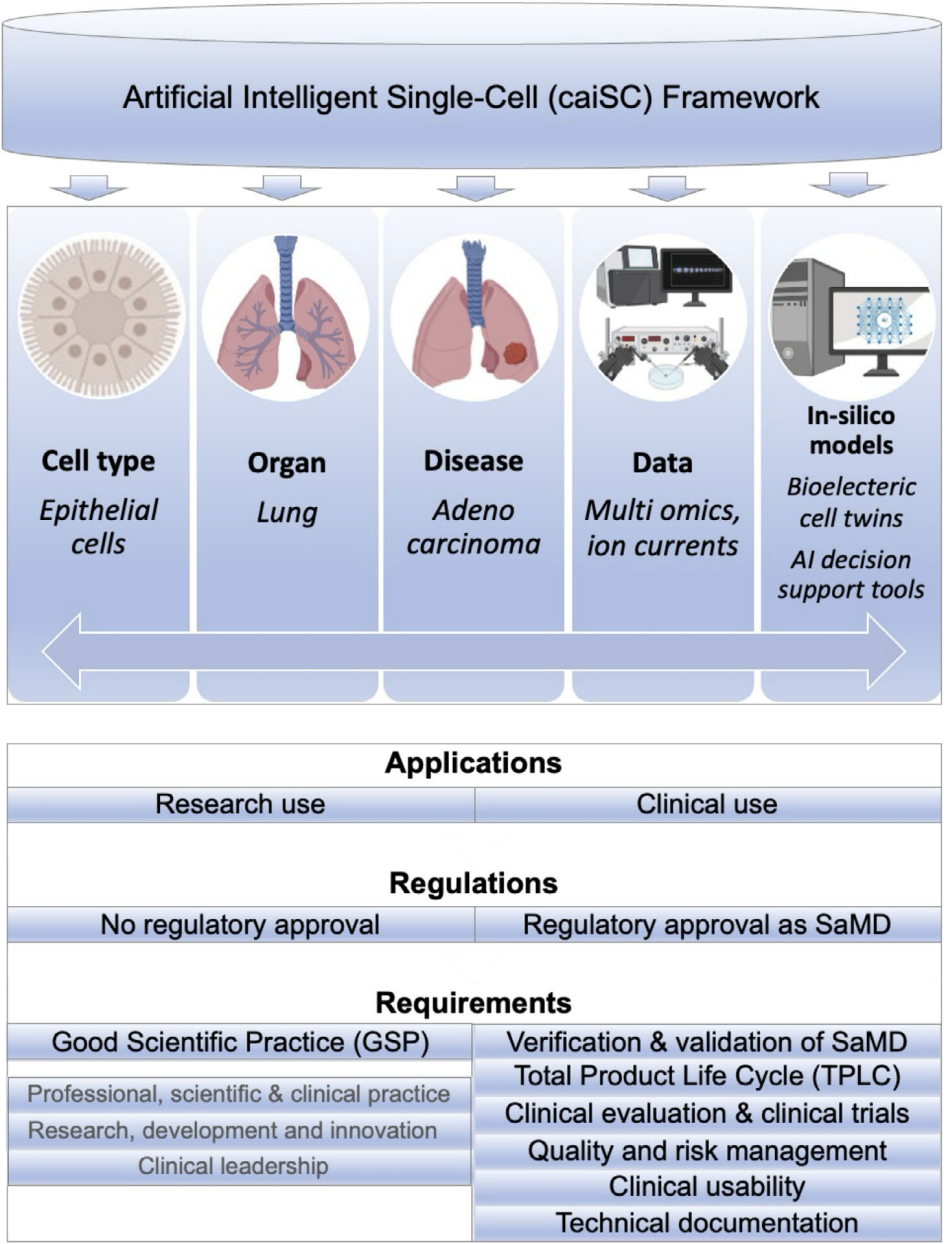
Extended clinical artificial intelligence single‐cell (caiSC) framework envisioned as a comprehensive knowledge base, integrating data from diverse cell types, organs, and diseases. Developed digital cell twins and machine learning/artificial intelligence (ML/AI)‐based clinical decision support models are applicable in both research and clinical settings; however, clinical use requires regulatory approval in line with international standards. The figure includes BioRender content.

The caiSC framework holds considerable promise for advancing single‐cell biology and its application in clinical decision‐making and precision medicine.^10^ While the system is not yet fully comprehensive, it provides valuable insights through its existing data and AI‐driven analyses. Further development efforts should prioritize expanding data integration, refining ML models, and enhancing the interpretability of its outputs for clinical application. Figure 2 illustrates an envisioned extension of the caiSC framework as a comprehensive knowledge base that is intended to transform decision‐making in clinical medicine.

The integration of digital twins and ML/AI‐based decision support models within the caiSC framework represents a pivotal advancement toward personalized medicine. These models could enhance predictive diagnostics and treatment optimization, and deepen our understanding of disease mechanisms. However, for the system to fully achieve its clinical potential, challenges related to data availability, computational resources, and regulatory approval must be addressed. With continued advancements, the caiSC framework has the potential to revolutionize healthcare by delivering more precise, personalized treatments and improving patient outcomes.

## AUTHOR CONTRIBUTIONS

Christian Baumgartner and Dagmar Brislinger conceived, wrote, and reviewed the article.

## ETHICS STATEMENT

Not applicable
